# Malocclusion complexity and orthodontic treatment need in children with autism spectrum disorder

**DOI:** 10.1007/s00784-022-04578-8

**Published:** 2022-06-15

**Authors:** Stephanie A. Meuffels, Anne Marie Kuijpers-Jagtman, Stephen T. H. Tjoa, Clarissa C. Bonifacio, Paola L. Carvajal Monroy

**Affiliations:** 1 Department of Pediatric Dentistry, Academic Centre for Dentistry Amsterdam (ACTA), Amsterdam, the Netherlands; 2 Department of Orthodontics, University Medical Center Groningen, University of Groningen, Groningen, the Netherlands; 3 Department of Orthodontics and Dentofacial Orthopedics, School of Dental Medicine/Medical Faculty, University of Bern, Bern, Switzerland; 4 Faculty of Dentistry, Universitas Indonesia, Jakarta, Indonesia; 5 Department of Oral and Maxillofacial Surgery, Special Dental Care and Orthodontics, Erasmus Medical Center, Rotterdam, the Netherlands

**Keywords:** Autism spectrum disorder, Child, Adolescent, Orthodontic care, Malocclusion, Index of Orthodontic Treatment Need, Needs assessment

## Abstract

**Objectives:**

This study aimed to investigate the malocclusion complexity and orthodontic treatment need among children with and without autism spectrum disorder (ASD) referred for orthodontic treatment by quantifying the Discrepancy Index (DI) and Index of Orthodontic Treatment Need (IOTN).

**Materials and methods:**

Dental records of 48 ASD and 49 non-ASD consecutive patients aged between 9 and 18 years (median age 13.0 years) referred for orthodontic treatment were reviewed and compared. The Discrepancy Index (DI) was quantified to determine the malocclusion complexity, and the Index of Orthodontic Treatment Need (IOTN), including the Dental Health Component (IOTN-DHC) and Aesthetic Component (IOTN-AC), was quantified to determine the orthodontic treatment need. Statistical analysis included descriptive analysis, Pearson chi-square tests, Fisher’s exact test, Mann–Whitney *U* tests, and several univariate and multivariate regression analyses. The statistical analysis used descriptive analysis, Pearson chi-square test, Fisher’s exact test, and multivariate logistic regression.

**Results:**

The results show that both malocclusion complexity (DI, *p* = 0.0010) and orthodontic treatment need (IOTN-DHC, *p* = 0.0025; IOTN-AC *p* = 0.0009) were significantly higher in children with ASD. Furthermore, children with ASD had a higher prevalence of increased overjet (*p* = .0016) and overbite (*p* = .031).

**Conclusions:**

Malocclusion complexity and orthodontic treatment need are statistically significantly higher among children with ASD than children without ASD, independent of age and sex.

**Clinical relevance:**

Children with autism may benefit from visits to a dental specialist (orthodontist) to prevent, to some extent, developing malocclusions from an early age.

## Introduction

Autism spectrum disorder (ASD) is a group of neurodevelopmental disorders with key features, including impaired social interaction and communication and restricted or repetitive behavioral stereotypes [1]. The prevalence in developed countries is estimated at 1.5% [2, 3].

Oral care for children with ASD can be challenging at home and the dental office. Next to the common problems such as communication and interaction problems, other autism-related factors are associated with non-cooperative behavior during the oral care process [4]. Therefore, children experience significant difficulties and barriers regarding oral care [5, 6]. Children with ASD do not exhibit particular oral anomalies related to the spectrum. However, they have a high risk for oral diseases such as caries, periodontal disease, dental trauma, parafunctions, and malocclusions [7, 8].

Children with ASD commonly display oversensitivity in and around the mouth, leading to extreme aversive responses to touch to different textures of food or objects placed in this area [9]. Food selectivity ranges between 46 and 89% in children with ASD [10, 11]. For this reason, their diet is often limited to a few soft, sticky, and sweet foods [12]. Moreover, parents and caregivers commonly use sweet snacks as a reward to reinforce behavior [13, 14]. Additionally, many of these children receive medications that may have adverse side effects on oral and gingival health, mainly if they contribute to gingival overgrowth and salivary dysfunction [15]. A diet high in simple sugars (mono- or disaccharides) together with a high intake frequency and the restricted protective mechanism of the saliva increases the risk of caries in these patients [14, 16].

The majority of children with ASD have poor oral hygiene, and almost all of them have gingivitis. Oversensitivity and delay in motor development compromise adequate oral hygiene [6, 17]. Also, aversion to the taste of toothpaste and the sensation of a toothbrush contribute to inadequate plaque control [7].

Cognitive and developmental delay, reduced motor coordination, and self-injurious behavior might explain why children with ASD are more prone to dental trauma [18–21]. In its turn, the high prevalence of parafunctions such as finger or thumb sucking, tongue pressing, lip biting, bruxism, and pica directly affects the development of the dentition and often leads to malocclusions [22–24]. Studies regarding the prevalence of malocclusion in children with ASD show contradictory results. Previous studies demonstrated a higher prevalence of overall malocclusion among children with ASD [23]. Other authors found no significant difference, but they did show certain malocclusion traits to be more common in the ASD population [25–27].

All in all, studies on malocclusions are scarce, and there is a lack of evidence on malocclusion complexity and orthodontic treatment need in children with ASD. Therefore, this study aims to evaluate malocclusion complexity by quantifying the Discrepancy Index (DI) and determining orthodontic treatment need by assessing the Index of Orthodontic Treatment Need (IOTN) in children with and without ASD. We hypothesize that the complexity of malocclusion and the need for orthodontic treatment are higher in children with ASD.

## Subjects and methods

This study was performed in line with the principles of the Declaration of Helsinki. Approval was granted by the Medical Ethical Committee of Erasmus Medical Center (date: 16–02-2021/No. 2021–0111).

### Population

**Fig. 1:**
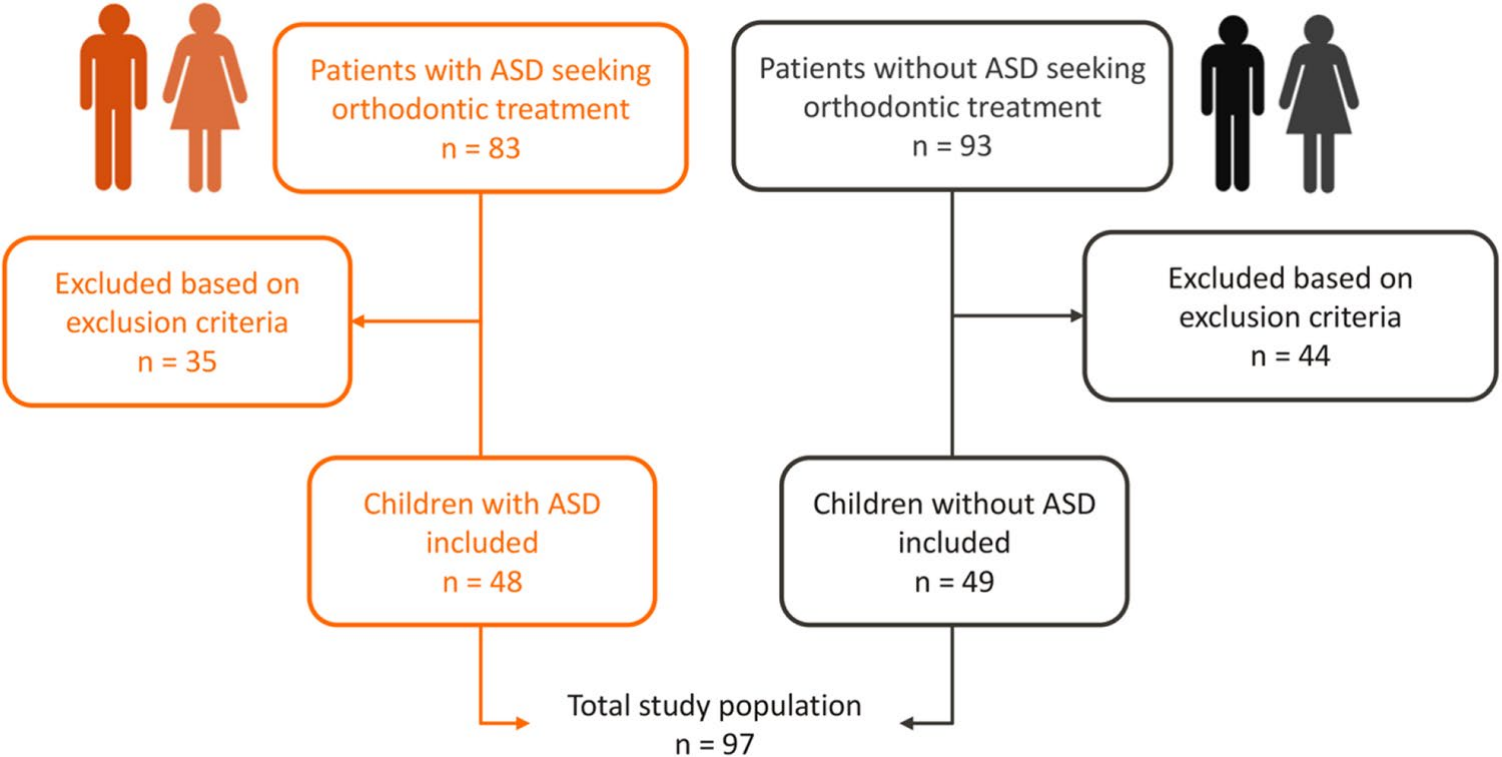
The population selection. *Note.* The flowchart presents the population selection of this study

The study includes children with and without autism referred to the same specialist in orthodontics between January 2018 and December 2020 (PCM-principal author). The study group and control comprised consecutive patients with ASD (*n* = 83, Fig. 1) and without ASD (*n* = 93, Fig. 1) referred for orthodontic treatment to Erasmus Medical Center / Sophia Children’s Hospital and a dental clinic, respectively. The principal author works full-time at Erasmus Medical Center / Sophia Children’s Hospital and only has a small (one-chair) non-hospital clinic consult 2 days per month, where average patients are referred for orthodontic treatment. It explains the small size of the control group over the 3 years’ time (*n* = 93, Fig. 1). The inclusion criteria for the study group were (1) children with a confirmed diagnosis of ASD, (2) aged between 9 and 18 years, and (3) patients in the mixed or permanent dentition. The eligibility criteria of the control group were (1) children without ASD, (2) aged between 9 and 18 years, and (3) with mixed or permanent dentition. Children with incomplete dental records or who presented with a congenital facial deformity, including any craniofacial anomaly or cleft lip and/or palate, were excluded.

### Study procedures

**Fig. 2:**
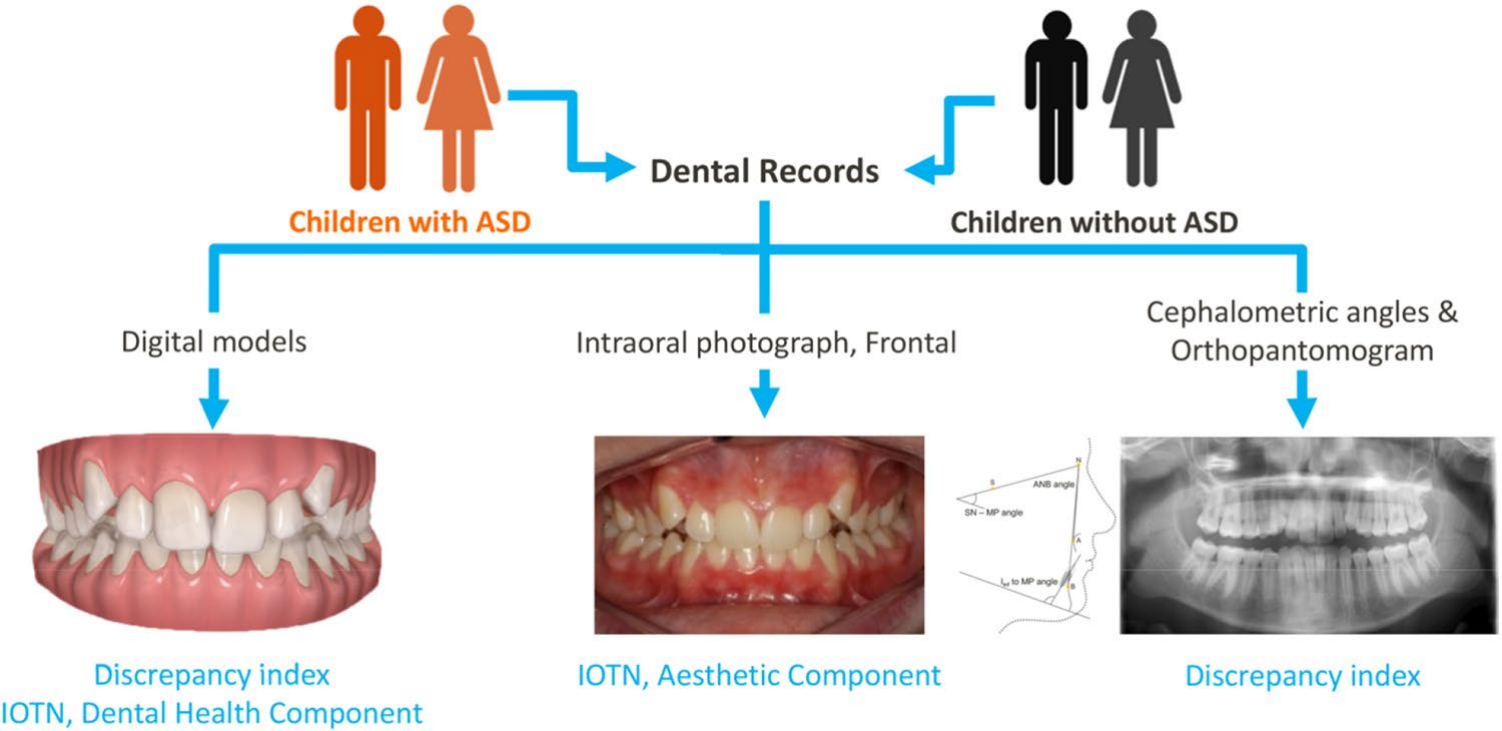
The study protocol. *Note.* The flowchart shows the study protocol: dental records of children with ASD and without ASD were analyzed by a reviewer blinded for the patient group. A digital dental model, a frontal intraoral photograph, an orthopantomogram, and cephalometric angles (ANB, SNB–MP, and *I*_nf_ to MP) were used to determine the Discrepancy Index (DI) and the Index of Orthodontic Treatment Need (IOTN)

One orthodontist performed the cephalometric analysis and collected all raw data. Variables such as age, gender, mental disability, comorbidity, and medication were registered. A senior dental student was trained and calibrated for the indices used in this study by an expert orthodontist. The trained student evaluated each child’s digital dental model, frontal intraoral photograph, orthopantomogram, and cephalometric angles (ANB, SN-MP, and *I*_inf_ to MP angle) without any previous medical information of the children to ensure the review was blind and unbiased (Fig. 2). The ICC estimate was obtained using a two-way mixed-effects model with an absolute agreement on multiple raters (*k* = 2) of scores of 20 patient records [28].

The Discrepancy Index (DI) was quantified to determine the malocclusion complexity, and the Index of Orthodontic Treatment Need (IOTN), including the Dental Health Component (IOTN-DHC) and Aesthetic Component (IOTN-AC), was scored to determine the orthodontic treatment need. In addition, malocclusion traits such as Angle’s classification (class I, class II, class III), overjet (measured on incisors, in millimeters), overbite (measured on incisors, in millimeters), tooth agenesis (excluding third molars), impactions, crossbites (anterior or posterior), and open bites (anterior or posterior) were documented.

#### Discrepancy Index (DI)

The Discrepancy Index provides an objective method to indicate the severity of pretreatment malocclusion and case complexity. The index is assessed using standard pretreatment orthodontic records, including dental models, an orthopantomogram, and a lateral skull radiograph. Analysis of the orthodontic records was based on the conceptual framework proposed by Cangialosi et al. [29].

#### Index of Orthodontic Treatment Need (IOTN)

The Index of Orthodontic Treatment Need (IOTN) is a commonly used index to determine the need of orthodontic treatment of a patient. This index consists of two components: a clinical dental component called the Dental Health Component (DHC) and an Aesthetic Component (AC). DHC and AC are recorded individually and are not combined. The orthodontic records were analyzed as previously proposed by Brook and Shaw [30]. The Dental Health Component provides a graded system from 1 to 5 (5 being the most severe) and determines the nature and severity of a patient’s malocclusion. A DHC score of 3 (along with an AC score of 6 or more) and a score of 4 or 5 indicate the need for orthodontic treatment. The Aesthetic Component is an illustrated 10-point scale used to score the aesthetic impairment of malocclusion based on a frontal intraoral photograph. An AC score of 5–7 indicates borderline need for orthodontic treatment, and an AC score of 8–10 indicates need for orthodontic treatment.

#### Statistical analysis

GraphPad Prism version 9 for MacOSX (GraphPad Software: www.graphpad.com) was used for data analysis. Descriptive analysis was performed to provide a distribution summary for all variables. IOTN-DHC, IOTN-AC, and malocclusion traits were compared between children with and without ASD using Fisher’s exact and chi-square tests. The normality of age and DI scores was assessed using the Shapiro–Wilk test. A Mann–Whitney *U* test was performed to compare median age and DI scores of children with and without ASD.

For Fisher’s exact and Mann–Whitney *U* tests, a post hoc power analysis was performed using G Power v. 3.1.9.7 for MacOSX [31] Power (1-β) was calculated as the function of alpha, the population effect size, and *N*. For the Mann–Whitney U test, the effect size was calculated using the online effect size calculator for a nonparametric test (www.psychometrica.de) [32].

A multivariate linear regression model was performed to evaluate the association between the DI score and ASD, adjusted for associated factors sex and age. Logistic regression analyses and odds ratios were calculated to examine whether ASD was associated with the likelihood of orthodontic treatment indication (IOTN-DHC score ≥ 4 and IOTN-AC score ≥ 8) and the likelihood of having an Angle class II or III molar relationship by including sex and age as associated factors. Statistically significant differences were detected at probability values of 0.05 or less.

### Community involvement

This research was led by other oral health care professionals and members from the workgroup autism-friendly oral care.

## Results

### Descriptive statistics

Records of 176 consecutive patients referred for orthodontic treatment were reviewed and compared. After applying the inclusion and exclusion criteria, ninety-seven children were included in this study, 48 children with ASD and 49 children without ASD. A flowchart of the population selection is shown in Fig. 1.

Table 1 presents an overview of the characteristics and distribution of malocclusion traits in the study population. The groups did not differ in age, and the median age was 13 years (range: 10–18 years). The distribution of sex was statistically significantly different between the ASD and non-ASD groups (*p* = 0.0003) as most children in the ASD group were boys (81.3%). Comorbidities were present in 30 children of the ASD group (62.5%). The most common comorbidities were intellectual disability (43.8%) and a developmental disorder (43.8%), followed by attention deficit hyperactivity disorder (ADHD) (14.6%) and epilepsy (10.4%). Twenty-four children (50%) from the ASD group used prescribed medication, whereas no children of the non-ASD group used medication.

**Table 1:** Distribution of characteristics and malocclusion traits in ASD and non-ASD children

Variables		ASD *N* = 48	Non-ASD *N* = 49	*p* value
		Median or number (%)	Median or number (%)	
Age (years)^a^		13.00	13.00	0.83
Sex^b^	Male	39 (81.3)	22 (44.9)	**0.0003**
	Female	9 (18.8)	27 (55.1)	
Comorbidity	Reported	30 (62.5)	0 (0)	
	Non reported	18 (37.5)	49 (100)	
Medication	Prescribed medication	24 (50)	0 (0)	
	No prescribed medication	24 (50)	49 (100)	
Angle class^b^	Class I	10 (20.8)	13 (26.5)	0.61
	Class II	34 (70.8)	34 (69.4)	
	Class III	4 (8.3)	2 (4.1)	
Overjet^c^	< 1 mm	12 (25.0)	6 (12.2)	**0.0016**
	1 to ≤ 3 mm	5 (10.4)	10 (20.4)	
	> 3 to ≤ 8 mm	15 (31.3)	29 (59.2)	
	> 8 mm	16 (33.3)	4 (8.2)	
Overbite^c^	≤ 1 mm	10 (20.8)	7 (14.3)	**0.031**
	> 1 to ≤ 3 mm	6 (12.5)	11 (22.4)	
	> 3 to ≤ 7 mm	17 (35.4)	26 (53.1)	
	Impinging or 100%	15 (31.3)	5 (10.2)	
Crossbite^b^	Anterior	14 (29.2)	6 (12.2)	0.57
	Posterior	22 (45.8)	14 (28.6)	
Open bite^b^	Anterior	11(22.9)	7 (14.3)	0.36
	Posterior	7 (14.6)	1 (2.0)	
Tooth agenesis^b^	Present	6 (12.5)	5 (10.2)	0.76
	Non-present	42 (87.5)	44 (89.8)	
Tooth impaction^b^	Present	8 (16.7)	4 (8.2)	0.23
	Non-present	40 (83.3)	45 (91.8)	

*N* = 97. Percentages appear in parentheses next to numbers. Mann–Whitney *U* (a), Fisher’s exact (b), and chi-square (c) tests were used to examine relations between ASD and these variables. Bold values denote statistical significance at the *p* > 0.05

The Angle classification showed a comparable prevalence of class II for the ASD (70.8%) and non-ASD (69.4%) groups. Class III malocclusion was present in 8.3% of children with ASD and 4.1% of children without ASD. Fisher’s exact test did not show any statistically significant difference in the Angle classification between both groups (*p* = 0.61).

More than one-third (33.3%) of the ASD group showed an overjet of > 8 mm, whereas this was the case in only 8.2% of the non-ASD group. Also, there was a higher prevalence of decreased overjet (< 1 mm) in the ASD group (25.0%), compared to the non-ASD group (12.2%). These differences were found to be statistically significant (*p* = 0.0016).

Looking at overbite, the children with ASD had a higher prevalence of decreased (< 1 mm) and extreme increase (impinging or 100%) of overbite compared to children without ASD. One-quarter (25%) of the children with ASD presented a decreased overbite (< 1 mm), whereas 14.8% of the non-ASD group presented this type of overbite. Only 10.2% of the non-ASD group had an impinging or complete overbite. This is significantly less than the 31.3% of children with ASD with this type of overbite. The co-currency between overbite and ASD was statistically significant (*p* = 0.031).

Although not statistically significant, a higher prevalence of crossbite (*p* = 0.57) and open bite (*p* = 0.36) was observed in children with ASD. Almost three-quarters (74.7%) of children with ASD had an anterior or posterior crossbite, compared to 40.8% without ASD. Open bite was present in 37.5% of the children with ASD. This was the case in 16.3% of the non-ASD group. The prevalence of tooth agenesis and tooth impaction was comparable between the groups (*p* = 0.76 and *p* = 0.23, respectively).

Table 2 presents the distribution of DI, IOTN-DHC, and IOTN-AC among the study population. The Shapiro–Wilk test indicated that the DI scores were not normally distributed (*p* = 0.047). Therefore, a Mann–Whitney *U* test was performed to evaluate differences in median DI score between the ASD and non-ASD groups. As shown in Table 2, this test indicated that DI scores were significantly higher in children with ASD compared to children without ASD (*p* = 0.0002).

**Table 2:** Distribution of DI, IOTN-DHC, and IOTN-AC in ASD and non-ASD children

Variables		ASD *N* = 48	Non-ASD *N* = 49	*p* value
		Median or number (%)	Median or number (%)	
DI score		27.50	20.0	**0.0002**
IOTN-DHC	No treatment need (score 0–3)	10 (20.8)	27 (55.1)	**0.0007**
	Treatment need (4–5)	38 (79.2)	22 (44.9)	
IOTN-AC	No treatment need (score 0–7)	34 (70.8)	47 (95.9)	**0.0009**
	Treatment need (score 8–10)	14 (29.2)	2 (4.1)	

*Abbreviations*: *DI*, Discrepancy Index; *IOTN-DHC*, Index of Orthodontic Treatment Need – Dental Health Component; *IOTN-AC*, Index of Orthodontic Treatment Need – Aesthetic Component

Percentages appear in parentheses next to numbers. Group differences were tested using Fisher’s exact tests for the variables IOTN-DHC and IOTN-AC. A Mann–Whitney *U* test was performed to evaluate differences in median DI score. Bold values denote statistical significance at the *p *> 0.05

The IOTN-DHC revealed that a substantial portion (79.2%) of the ASD group had an indicated treatment need, whereas this was the case in 44.9% of the non-ASD group. Only 20.8% of the ASD group had no indicated treatment need, compared to 55.1% of the non-ASD group. The differences in IOTN-DHC between ASD and non-ASD were statistically significant (*p* = 0.0007).

According to the IOTN-AC, almost one-third (29.2%) of the ASD group presented a score of 8–10, indicating treatment need. This was the case in only 4.1% of the non-ASD group. The non-ASD group presented higher percentages (95.9%) of no treatment indication when compared to the ASD group (70.8%). The relation between ASD and IOTN-AC was statistically significant (*p* = 0.0009).

### Post hoc power analysis

The post hoc power analysis showed an effect size = 0.80 and power of 1-β = 0.96 (sample size = 97, *α* = 0.05), indicating that the number of participants was appropriate.

### Rater’s reliability

An intraclass correlation coefficient (ICC) of 0.9 was calculated, indicating an excellent intra- and interrater reliability.

### Analysis

#### Malocclusion complexity (DI score)

Table 3 presents univariate and multivariate linear regression analyses to evaluate the association between DI score and ASD by including sex and age as associated factors. The unadjusted analysis determined that in children with ASD, an increase in DI score of 11.45 is predicted (*p* < 0.0001; 95% CI 6.09 to 16.82). The model explains 15.90% (*R*^2^) of the variability in the DI score. Interestingly, after adjusting the model for associated factors sex and age, a significant increase in DI score of 9.78 is predicted in children with ASD (*p* = 0.0010; 95% CI 4.05–15.51), indicating greater malocclusion complexity. There was no evidence that demographic factors sex (*p* = 0.15) and age (*p* = 0.20) were statistically significantly associated with DI score. The *R*^2^ estimate of the adjusted model was found to be 19.49%, indicating that the model with ASD, sex, and age explains 19.49% of the total variation in DI score.

**Table 3:** Univariate and multivariate linear regression analyses of DI score and associated factors

	Unadjusted (*R*^2^ = 0.15)				Adjusted (*R*^2^ = 0.19)			
	*B*	SE	*p* value	95% CI	*B*	SE	*p* value	95% CI
Patient status (non-ASD vs. ASD)	11.45	2.70	** < 0.0001**	6.09–16.82	9.78	2.88	**0.0010**	4.05–15.51
Sex (female vs. male)	8.46	2.92	**0.0047**	2.65–14.26	4.39	2.99	0.15	−1.55 to 10.33
Age (years)	1.03	0.75	0.17	–0.46 to 2.51	0.90	0.69	0.20	–0.47 to 2.26

*Abbreviations*: *R*.^2^, *R*-squared, coefficient of determination; *B*, unstandardized coefficient; *SE*, standard error; *CI*, confidence interval. Bold values denote statistical significance at the *p *> 0.05

#### Orthodontic treatment need (IOTN-DHC and IOTN-AC)

Univariate and multivariate logistic regression analyses were conducted to examine whether ASD was associated with the likelihood of orthodontic treatment need (IOTN-DHC score ≥ 4, IOTN-AC score ≥ 8, and IOTN-DHC score 3 combined with IOTN-AC score 6). The substantial difference in sex distribution between the ASD and the non-ASD groups was controlled by including sex and age as associated factors in the regression analyses. The results, as shown in Table 4, revealed that children with ASD were 4.44 times more likely to have an orthodontic treatment need (IOTN-DHC score ≥ 4) than children without ASD, independently of their demographic factors sex and age (OR, 4.44; 95% CI, 1.74–12.15; *p* = 0.0025). As presented in Table 4, the adjusted logistic regression analysis for IOTN-AC score revealed that the odds of having an orthodontic treatment indication (IOTN-AC score ≥ 8) are 8.59 times higher in children with ASD compared to children without ASD (OR, 8.59; 95% CI, 2.08–59.19; *p* = 0.0083). Demographic factors sex (*p* = 0.56) and age (*p* = 0.42) were not statistically significantly associated with orthodontic treatment need.

**Table 4:** Univariate and multivariate logistic regression analyses of orthodontic treatment indication and associated factors

	Unadjusted			Adjusted		
	OR	95% CI	*p* value	OR	95% CI	*p* value
IOTN-DHC score 3 combined with IOTN-AC score 6, or IOTN-DHC ≥ 4						
Patient status (non-ASD vs. ASD)	4.30	1.80–10.89	**0.0014**	4.44	1.74–12.15	**0.0025**
Sex (female vs. male)	1.64	0.70–3.85	0.25	1.02	0.38–2.64	0.97
Age (years)	0.88	0.71–1.08	0.22	0.86	0.68–1.07	0.17
IOTN-AC score ≥ 8						
Patient status (non-ASD vs. ASD)	9.68	2.49–64.24	**0.0040**	8.59	2.08–59.19	**0.0083**
Sex (female vs. male)	2.98	0.88–13.73	0.11	1.55	0.38–7.83	0.56
Age (years)	1.11	0.84–1.44	0.46	1.13	0.83–1.54	0.42

*Abbreviations*: *OR*, odds ratio; *CI*, confidence interval. Bold values denote statistical significance at the* p *> 0.05

## Discussion

This study aimed to determine malocclusion complexity and orthodontic treatment need in children with ASD referred for orthodontic treatment. Outcome parameters were DI to determine malocclusion complexity and IOTN-DHC/IOTN-AC to determine orthodontic treatment needs. Our results demonstrate that both malocclusion complexity and orthodontic treatment need are statistically significantly higher in children with ASD than children without ASD, independent of age and sex. Furthermore, increased overjet and overbite were more common in children with ASD than those without ASD.

As mentioned before, studies on malocclusion traits in children with ASD are scarce. In line with a previous study, our results demonstrate that the prevalence of malocclusion is statistically significantly higher in children with ASD than that in children without ASD [23]. In contrast, other studies found no significant difference in the overall prevalence of malocclusion in children with ASD [25, 33, 34]. However, all mentioned studies found a higher prevalence for at least one malocclusion trait in children with ASD. For instance, several studies showed a significantly increased overjet among children with ASD [23, 25, 33].

While some research has been carried out to study malocclusion traits in children with ASD, this study is the first comprehensive investigation of malocclusion complexity in this population. The findings of the linear regression analysis predicted an increase in DI score of 9.78 in children with ASD compared to children without ASD, independent of demographic factors sex and age. An increase in DI score is associated with greater malocclusion complexity, resulting in a more substantial orthodontic treatment challenge [29]. Therefore, the current data contribute to our understanding of orthodontic case complexity in children with ASD.

The results for the IOTN-DHC and IOTN-AC scores have shown that children with ASD were respectively 4.44 and 8.52 times more likely to have orthodontic treatment need than children without ASD. A possible explanation for these results may be the high prevalence of parafunctions in this population, such as finger or thumb sucking, tongue pressing, and lip/object biting [22, 24, 26, 35, 36].

Unfortunately, only a few studies on orthodontic treatment need in children with ASD have been published. A recent study among Indonesian children with ASD found that 20% of the tested children showed an IOTN-DHC score ≥ 4 (*n* = 4), indicating orthodontic treatment need [37]. However, these findings may be compromised by the small sample size (*n* = 20), the lack of comparison with a control group, and the lack of statistical tests to provide information about the significance of their results. Another study used the Dental Aesthetic Index (DAI) to determine orthodontic treatment needs among children with ASD in Thailand [34]. These authors did not find a statistically significant difference in DAI between children with and without ASD. The discrepancy with the current study could be attributed to essential differences between the IOTN and the DAI, both widely used indexes to assess orthodontic treatment need. The IOTN-DHC identifies occlusal characteristics which are functionally disadvantageous, whereas the DAI consists of a continuous scale and prioritizes esthetic features [38]. Although these authors did not find a statistically significant difference in DAI between children with and without ASD, their results show higher percentages in class II molar relationship, reverse overjet, spacing, open bites, and missing teeth among children with ASD [34].

An important discussion point is a statistically significant difference in the distribution of sex between the ASD and non-ASD groups in this study, as most children in the ASD group were boys (81.3%). Consequently, multivariate regression analyses were conducted to avoid interference of confounding variables. Nevertheless, the prevalence of ASD is higher among boys. Only 1 in 4 children with ASD is a girl [39]. Therefore, our study group can be considered a close representation of the ASD population.

The sample size in this study was 97 children. We performed a post hoc power analysis, and the sample size proved to be adequate. A point of concern might be the sample selection in two different orthodontic clinics. The Dutch health care system is based on access to care for all, medical insurance being compulsory for all residents of the Netherlands [40]. The central government determines the fees; and therefore, dental and orthodontic treatment fees are the same in all clinics. Most orthodontic practices in the Netherlands are busy high-volume multi-chair offices. In contrast, the clinic where the ASD patients were treated has fewer chairs and reserves more time per patient. The department has an excellent reputation for its affinity with ASD patients, and for this reason, we have a high number of children with ASD. Children with ASD are not referred to Erasmus Medical Center / Sophia Children’s Hospital because of the severity of the orthodontic diagnosis and/or complexity of the treatment but because of their perceived communication and interaction problems or non-cooperative behavior during treatment. We consider that both groups are as similar as possible, except for the presence or absence of an autism spectrum disorder under study. Therefore, a blind assessment of study models, radiographs, and photographs was crucial in our study. It helps minimize systematic differences in how outcomes are ascertained.

Since both groups comprise patients referred for orthodontic treatment, the observed outcomes might be overestimated. Hence, the generalizability of the results is subject to limitations. Likewise, being limited by the cross-sectional nature of this study, no judgment on a cause-effect relationship can be implied. Nevertheless, the literature lacks studies assessing malocclusion complexity and orthodontic treatment need in children with ASD, and the present study is one of the largest reported to date regarding these two research questions. However, there are still many unanswered questions. Further research should focus on determining causative factors involved in the increased malocclusion complexity, and orthodontic treatment need in children with ASD, helping us establish a greater degree of understanding on this subject.

Notwithstanding its limitations, this study certainly provides insights into this critical topic. Orthodontic care for children with ASD can undoubtedly be a challenge due to impaired social interaction and anxiety for dental treatment [5, 41, 42]. While these children often have barriers to oral care, early initiation of oral health education in children with ASD has shown to have a positive effect on oral hygiene [43]. Therefore, the findings of this study have important implications for considering and developing an interceptive orthodontic approach at an early age for children with ASD. Here, adherence to therapy may increase since a trusting relationship between the child and the dentist and orthodontist can be facilitated earlier [44, 45], limiting the extent of orthodontic treatment at late years. Future studies might explore this approach.

In conclusion, the results of this study show that malocclusion complexity and orthodontic treatment need are statistically significantly higher among children with ASD than those among children without ASD, independent of sex and age. Consequently, children with autism may benefit from visits to a dental specialist (orthodontist) to prevent, to some extent, developing malocclusions from an early age.
